# Glucagon-like-peptide-1 (GLP-1) receptor agonist use and the risk of pulmonary aspiration in patients undergoing surgery

**DOI:** 10.1097/JS9.0000000000002425

**Published:** 2025-05-12

**Authors:** Jason D. Wright, Ling Chen, Xiao Xu, Chin Hur, Koji Matsuo, Elena B. Elkin, Dawn L. Hershman

**Affiliations:** a Division of Gynecologic Oncology, Department of Obstetrics and Gynecology, Columbia University College of Physicians and Surgeons, New York, New York State, USA; b Herbert Irving Comprehensive Cancer Center, New York, New York State, USA; c New York-Presbyterian Hospital, New York, New York State, USA; d Department of Medicine, Columbia University College of Physicians and Surgeons, New York, New York State, USA; e Department of Obstetrics and Gynecology, University of Southern California, Los Angeles, California; f Joseph L. Mailman School of Public Health, Columbia University, New York, New York, USA

**Keywords:** aspiration, GLP-1 receptor agonist, surgery

## Abstract

Glucagon-like-peptide-1 (GLP-1) receptor agonists are approved for the treatment of type II diabetes mellitus and obesity. These agents slow gastric emptying and may increase the risk of pulmonary aspiration, particularly in patients undergoing elective surgery. We used a claims database to examine the association between perioperative GLP-1 receptor agonist use and the risk of pulmonary aspiration. A total of 392 065 patients including 15 745 (4.0%) who used GLP-1 receptor agonist who underwent elective surgery were identified. The rate of pulmonary aspiration was 0.8% in those who received GLP-1 receptor agonists versus 0.7% in those who had not (*P* = 0.61). After adjusting for other risk factors for aspiration, there was no association between GLP-1 agonist use and aspiration (OR = 1.07; 95% CI, 0.85–1.34).

## Introduction

Glucagon-like-peptide-1 (GLP-1) receptor agonists are approved for the treatment of type II diabetes mellitus and obesity. These agents slow gastric emptying, inhibit release of glucagon, and stimulate insulin production^[^1^]^. As GLP-1 receptor agonists slow gastric emptying, prior studies of patients undergoing esophagogastroduodenoscopy have shown that the drugs increase residual gastric contents^[^2,3^]^. Whether this results in an increased risk of pulmonary aspiration, particularly for elective surgery, remains uncertain.^[^4-6^]^ However, given the theoretic concern, highly publicized guidance has recommended holding these agents in patients undergoing surgery^[^1^]^. The objective of our study was to determine the association between use of GLP-1 receptor agonists and pulmonary aspiration among patients undergoing abdominal and thoracic surgery.

**HIGHLIGHTS**

- The use of GLP-1 receptor agonists is increasing among patients undergoing surgery.
- The rate of pulmonary aspiration is 0.8% among GLP-1 receptor agonist users.
- There is no increased risk of aspiration associated with GLP-1 receptor agonist use.

## Methods

The MarketScan Commercial Database was used to identify patients >18 years of age with diabetes mellitus or overweight or obesity who underwent abdominal or thoracic surgical procedures including coronary artery bypass grafting (CABG), lobectomy, pancreatectomy, thyroidectomy, colectomy, cholecystectomy, appendectomy, nephrectomy, cystectomy, hysterectomy or prostatectomy from 2015 to 2022. The cohort was limited to patients with insurance and prescription drug benefits from 3 months before through 3 months after surgery.

Patients based on receipt of GLP-1 receptor agonists including dulaglutide, exenatide, liraglutide, lixisenatide, or semaglutide within 30 days prior to the procedure. Those who had received a GLP-1 agonist but had their last prescription >30 days prior to surgery and obstetric patients were excluded. The primary outcome was aspiration pneumonia within 7 days of surgery.

**Figure 1.**
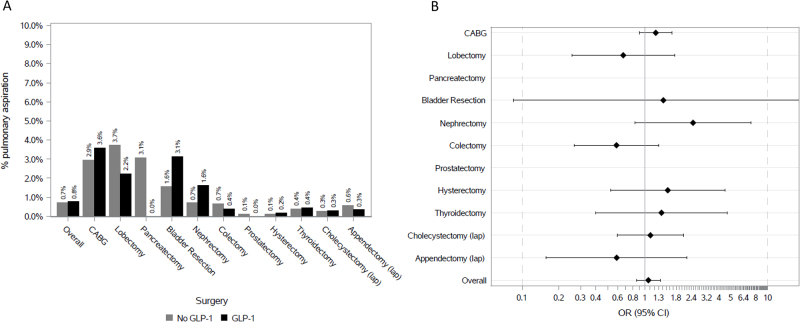
(A) Absolute rate of pulmonary aspiration stratified by use of GLP-1 agonist in the propensity score matched cohort. (B) Odds ratio of pulmonary aspiration stratified by GLP-1 agonist use in the propensity score matched cohort. Odds ratio for pulmonary aspiration estimated in the propensity matched cohort after adjustment for any of the additional risk factors including dysphagia, dyskinesia, esophageal stricture, esophageal cancer, esophagectomy, enteral feeding, seizure, multiple sclerosis, Parkinson’s disease, dementia, stroke, intracerebral hemorrhage, gastroesophageal reflux disease, hiatal hernia, and chronic obstructive pulmonary disease.

Trends in GLP-1 agonist use were assessed using Cochran-Armitage test. A propensity score (PS) matching analysis was used to estimate the predicted probability of receiving GLP-1 agonist as a function of baseline factors, diabetes mellitus, and degree of overweight and obesity (Table 1). A greedy nearest neighborhood matching using the standard deviation of the logit of the PS was used to generate a 2 (no GLP-1 agonist) to 1 (GLP-1 agonist) matched cohort. Standardized mean differences were calculated to measure balance diagnostics. After PS matching, logistic regression models adjusted for additional risk factors for aspiration were developed to estimate the risk of pulmonary aspiration (Fig. 1). Analyses for the overall sample and for each individual surgery were performed. Analyses were performed using SAS studio. A *P* < 0.05 was considered statistically significant.

**Table 1:** Baseline characteristics of the cohort, before and after 2:1 propensity score matching

	Crude					Propensity Score Matched			
	No GLP-1 Agonist		GLP-1 Agonist			No GLP-1 Agonist		GLP-1 Agonist		
	N	(%)	N	(%)	*StdDiff*	N	(%)	N	(%)	*StdDiff*
*All*	376 320	(96.0)	15 745	(4.0)		31 488	(66.7)	15 744	(33.3)	
*Age, in years*					0.28					0.00
18-49	162 993	(43.3)	4827	(30.7)		9716	(30.9)	4827	(30.7)	
50-59	105 201	(28.0)	5680	(36.1)		11 455	(36.4)	5679	(36.1)	
60-69	74 122	(19.7)	4096	(26.0)		8148	(25.9)	4096	(26.0)	
≥70	34 004	(9.0)	1142	(7.3)		2169	(6.9)	1142	(7.3)	
*Sex*					0.08					0.00
Male	131 072	(34.8)	6085	(38.6)		12 102	(38.4)	6084	(38.6)	
Female	245 248	(65.2)	9660	(61.4)		19 386	(61.6)	9660	(61.4)	
*Metropolitan statistical area*					0.12					0.06
Non-MSA	56 671	(15.1)	2574	(16.3)		4881	(15.5)	2573	(16.3)	
MSA	271 201	(72.1)	10 522	(66.8)		21 603	(68.6)	10 522	(66.8)	
Unknown	48 448	(12.9)	2649	(16.8)		5004	(15.9)	2649	(16.8)	
*Region*					0.08					0.05
Northeast	48 446	(12.9)	2068	(13.1)		4021	(12.8)	2067	(13.1)	
North central	98 723	(26.2)	3917	(24.9)		7707	(24.5)	3917	(24.9)	
South	186 800	(49.6)	8401	(53.4)		17 216	(54.7)	8401	(53.4)	
West	42 351	(11.3)	1359	(8.6)		2544	(8.1)	1359	(8.6)	
*Diabetes mellitus and obesity*					1.38					0.03
Overweight/obesity no diabetes	236 579	(62.9)	1431	(9.1)		2866	(9.1)	1431	(9.1)	
Diabetes and non-obesity	87 949	(23.4)	7390	(46.9)		15 082	(47.9)	7390	(46.9)	
Diabetes and obesity class I/II/unspecified	27 169	(7.2)	3366	(21.4)		6670	(21.2)	3366	(21.4)	
Diabetes and obesity class III	24 623	(6.5)	3558	(22.6)		6870	(21.8)	3557	(22.6)	
*Comorbidity*					0.25					0.00
0-1	174 258	(46.3)	5354	(34.0)		10 680	(33.9)	5354	(34.0)	
2	87 863	(23.3)	4269	(27.1)		8486	(26.9)	4269	(27.1)	
≥3	114 199	(30.3)	6122	(38.9)		12 322	(39.1)	6121	(38.9)	
*Year of surgery*										
Mean (SD)	2018	(2)	2019	(2)	0.42	2019	(2)	2019	(2)	0.01
2015	52 407	(13.9)	1210	(7.7)		2412	(7.7)	1210	(7.7)	
2016	57 497	(15.3)	1477	(9.4)		2964	(9.4)	1477	(9.4)	
2017	54 749	(14.5)	1719	(10.9)		3437	(10.9)	1719	(10.9)	
2018	52 816	(14.0)	1925	(12.2)		3926	(12.5)	1925	(12.2)	
2019	50 002	(13.3)	2171	(13.8)		4394	(14.0)	2171	(13.8)	
2020	37 217	(9.9)	1953	(12.4)		3875	(12.3)	1953	(12.4)	
2021	40 952	(10.9)	2720	(17.3)		5406	(17.2)	2719	(17.3)	
2022	30 680	(8.2)	2570	(16.3)		5074	(16.1)	2570	(16.3)	
*Surgery*					—					—
CABG	28 520	(7.6)	1869	(11.9)		3738	(11.9)	1869	(11.9)	
Lobectomy	7739	(2.1)	269	(1.7)		538	(1.7)	269	(1.7)	
Pancreatectomy	2793	(0.7)	65	(0.4)		130	(0.4)	65	(0.4)	
Bladder resection	1227	(0.3)	33	(0.2)		64	(0.2)	32	(0.2)	
Nephrectomy	9357	(2.5)	498	(3.2)		996	(3.2)	498	(3.2)	
Colectomy	51 069	(13.6)	2036	(12.9)		4072	(12.9)	2036	(12.9)	
Prostatectomy	12 133	(3.2)	452	(2.9)		904	(2.9)	452	(2.9)	
Hysterectomy	85 752	(22.8)	3293	(20.9)		6586	(20.9)	3293	(20.9)	
Thyroidectomy	22 011	(5.8)	1130	(7.2)		2260	(7.2)	1130	(7.2)	
Cholecystectomy (lap)	130 621	(34.7)	5238	(33.3)		10 476	(33.3)	5238	(33.3)	
Appendectomy (lap)	25 098	(6.7)	862	(5.5)		1724	(5.5)	862	(5.5)	

Abbreviations: StdDiff, standardized difference; MSA, metropolitan statistical area; SD, standard deviation.

Propensity score model included age, sex, year of surgery, metropolitan statistical area, region, Elixhauser comorbidity score (removing diabetes and obesity), and diabetes mellitus and obesity, and stratified by type of surgery.

## Results

A total of 392 065 patients including 15 745 (4.0%) who used GLP-1 receptor agonists and 376 320 (96.0%) who did not were identified (Table 1). GLP-1 agonist use rose from 2.3% in 2015 to 7.7% in 2022 (*P* < 0.001). After PS matching, the cohort was well balanced.

The rate of pulmonary aspiration was 0.8% in those who received GLP-1 receptor agonists versus 0.7% in those who had not (*P* = 0.61). After adjusting for other risk factors for aspiration, there was no association between GLP-1 agonist use and aspiration (OR = 1.07; 95% CI, 0.85–1.34). Similarly, there was no association between GLP-1 agonist use and aspiration for any of the individual procedures (Fig. 1).

## Discussion

Among patients treated with GLP-1 receptor agonists undergoing surgery, we noted no increased risk of pulmonary aspiration. Despite limited data, the risk of pulmonary aspiration associated with GLP-1 receptor agonist use has prompted clinical guidance recommending that these agents be held prior to elective surgery^[^1^]^. These recommendations have been widely implemented and may delay surgical procedures and necessitate changes in perioperative antidiabetic therapy.

While our sample was large, we cannot exclude the possibility that GLP-1 agonists are associated with a very small increased risk of aspiration. Additionally, there may be undercapture of use of these agents or pulmonary aspiration. Despite these limitations, our data are reassuring in that there appears to be no increased risk of pulmonary aspiration among GLP-1 receptor agonist users undergoing surgery.

## Data Availability

Data available from commercial source.
